# Investigating the Protective Mechanisms of Ginseng-Natto Composite Fermentation Products in Alzheimer’s Disease: A Gut Microbiota and Metabolomic Approach

**DOI:** 10.3390/ph19010123

**Published:** 2026-01-10

**Authors:** Zhimeng Li, He Wang, Huiyang Yuan, Yue Zhang, Bo Yang, Guoxin Ji, Zhuangzhuang Yao, Mingfang Kuang, Xian Wu, Shumin Wang, Huan Wang

**Affiliations:** 1 College of Pharmacy, Changchun University of Chinese Medicine, Changchun 130117, China; lzm2084@163.com (Z.L.); 15044100183@163.com (H.W.); 15104468789@163.com (H.Y.); yangbo0623@126.com (B.Y.); 17843099892@163.com (G.J.); 13461743039@163.com (Z.Y.); 18284640941@163.com (M.K.); 15043926050@163.com (X.W.); 2 Department of Scientific Research, Jilin Normal University, Siping 136000, China; jlnukyczy@jlnu.edu.cn; 3 Ginseng Scientific Research Institute, Changchun University of Chinese Medicine, Changchun 130117, China

**Keywords:** Ginseng-Natto composite fermentation products, Alzheimer’s disease, gut microbiota, metabolites, gut–brain axis, blood–brain barrier

## Abstract

**Background:** Alzheimer’s disease (AD), a progressive brain disorder, is the most common form of dementia and necessitates the development of effective intervention strategies. Ginseng-Natto composite fermentation products (GN) have demonstrated beneficial bioactivities in mouse models of AD; however, the underlying mechanism of action through which GN ameliorates AD requires further elucidation. **Methods:** Mice received daily intragastric administration of low- or high-dose GN for 4 weeks, followed by intraperitoneal injection of scopolamine to induce the AD model. The pharmacological effects of GN were systematically evaluated using the Morris water maze test, ELISA, and H&E staining. To further investigate the underlying mechanisms, 16S rRNA gene sequencing and metabolomics were employed to analyze the regulatory effects of GN on the gut–brain axis. Additionally, Western blotting was performed to assess the impact of GN on blood–brain barrier (BBB) integrity. **Results:** GN intervention significantly ameliorated cognitive deficits and attenuated neuropathological injury in AD mice, restoring the brain levels of acetylcholine (ACh), acetylcholinesterase (AChE), superoxide dismutase (SOD), malondialdehyde (MDA), glutathione peroxidase (GSH-Px), interleukin-6 (IL-6), and tumor necrosis factor-α (TNF-α) to normal ranges. GN reshaped the gut microbiota by promoting beneficial bacteria and inhibiting pro-inflammatory strains. It also regulated key metabolic pathways related to amino acid and unsaturated fatty acid metabolism. This metabolic remodeling restored the compromised BBB integrity by upregulating tight junction proteins (ZO-1, Occludin and Claudin-1). **Conclusions:** Our findings demonstrate that GN ameliorates AD through a gut-to-brain pathway, mediated by reshaping the microbiota-metabolite axis and repairing the BBB. Thus, GN may represent a promising intervention candidate for AD.

## 1. Introduction

Alzheimer’s disease (AD) is a progressive neurodegenerative disorder characterized by an insidious onset and represents the leading cause of dementia, accounting for 60–70% of cases [1]. It predominantly affects the elderly, particularly those with advanced age, a genetic predisposition, and concomitant cardiovascular and cerebrovascular risk factors [2,3]. The disease burden is intensifying, with global prevalence projected to surge from over 55 million to 139 million by 2050 [4]. Clinically, patients typically present with a progressive and irreversible decline in cognitive function. This condition severely compromises their quality of life and imposes a substantial burden on both families and society [5]. However, current pharmacotherapies, primarily cholinesterase inhibitors and NMDA receptor antagonists, offer only symptomatic relief and fail to halt disease progression [6]. Given these limitations associated with existing single-target agents, there is an urgent need for novel intervention strategies that can provide synergistic neuroprotective effects. In this context, Ginseng-Natto composite fermentation products (GN), a novel fermented product, emerges as a promising candidate.

Ginseng-Natto composite fermentation products (GN) are a novel edible fermented product derived from the fermentation of ginseng and soybeans with *Bacillus subtilis*. Numerous active components in GN have been demonstrated to possess significant biological activities, including neuroprotection, anti-amyloidogenic effects, and promotion of fibrinolysis [7,8]. He et al. [9] demonstrated that total ginsenosides, the primary active components of ginseng, are beneficial in AD treatment by enhancing spatial learning and memory in AD mice and reducing Aβ accumulation in the cortex and hippocampus. Similarly, the team led by Tanikawa reported that the oral administration of nattokinase in an AD rat model increased the levels of free-form Aβ in the cerebrospinal fluid and ameliorated the accumulation of aluminum and amyloid plaques in the brain [10]. Furthermore, Essawy et al. [11] found that supplementation with soy isoflavones significantly inhibited oxidative stress and neuroinflammation in colchicine-treated rats and reversed the overproduction of Aβ_1–42_ and pro-inflammatory cytokines in the brain.

The primary pathological hallmarks of AD are the deposition of Aβ into senile plaques and the hyperphosphorylation of Tau protein, leading to neurofibrillary tangles, which ultimately result in neuronal loss in key brain regions such as the hippocampus and cortex [12,13]. However, the gut–brain axis theory offers a new perspective, establishing the gut microbiota and its metabolites as key players profoundly implicated in the pathological progression of AD [14,15]. Consequently, the gut microbiota is regarded as a modifiable therapeutic target; by modulating specific microbial ratios or reshaping the community structure, it is possible to systemically regulate the host’s immune, metabolic, and neuroinflammatory states [16]. Although alterations in the gut microbiota have been consistently observed in AD patients and animal models, with certain taxa becoming key foci in pathogenesis research, the precise correlation network between gut microbes and host metabolites remains unclear [17,18]. Therefore, an integrated analysis of microbiomics and metabolomics is crucial for unraveling the underlying mechanisms of AD and developing novel intervention strategies.

This study systematically evaluated the beneficial effects of GN on the pathophysiology of AD and elucidated its underlying molecular mechanisms. We first quantified the main components of GN using High-Performance Liquid Chromatography (HPLC) and an agarose-fibrin plate assay. Subsequently, the Morris Water Maze (MWM) test, Enzyme-linked immunosorbent assays (ELISA), and Hematoxylin and Eosin (H&E) staining were employed to assess the ameliorative effects of GN on cognitive function, pathological indicators, and neuronal damage in AD mice. To elucidate its underlying mechanisms, this study combined 16S rRNA gene sequencing and metabolomics analysis to clarify the regulatory effects of GN on the gut microbiota–metabolism axis. Furthermore, Western blotting was used to verify its protective effect on the expression of blood–brain barrier (BBB) tight junction proteins. Collectively, these findings provide a solid scientific basis for the clinical application of GN to ameliorate AD.

## 2. Results

### 2.1. Contents of Major Bioactive Constituents in GN

**Figure 1:**
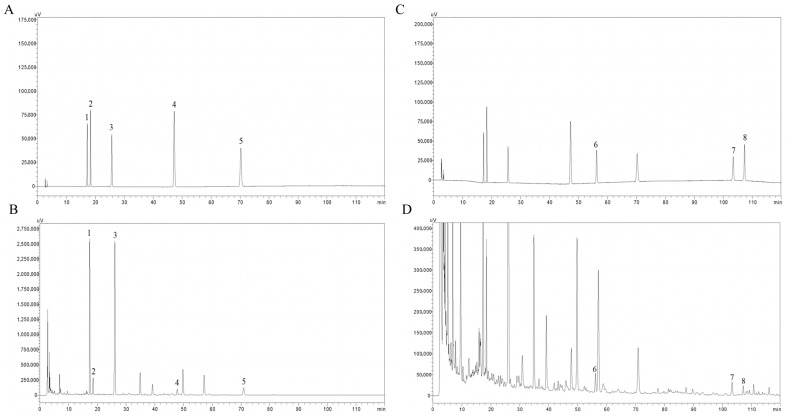
HPLC peaks of the mixed standard and the GN sample. (**A**,**B**) HPLC peaks of the mixed standard and the GN sample at 254 nm. (**C**,**D**) HPLC peaks of the mixed standard and the GN sample at 203 nm. (1. Daidzin, 2. Glycitin, 3. Genistin, 4. Daidzein, 5. Genistein, 6. Ginsenoside Rg1, 7. Ginsenoside Rb1, 8. Ginsenoside Rc).

The major bioactive constituents in GN, including nattokinase, soy isoflavones (daidzin, glycitin, genistin, daidzein, and genistein), and ginsenosides (ginsenoside Rg1, Rb1, and Rc) are summarized in Table 1. The HPLC chromatograms of the mixed standards and the GN sample are presented in Figure 1.

### 2.2. Effects of GN on Learning and Memory Impairment of AD Mice

**Figure 2:**
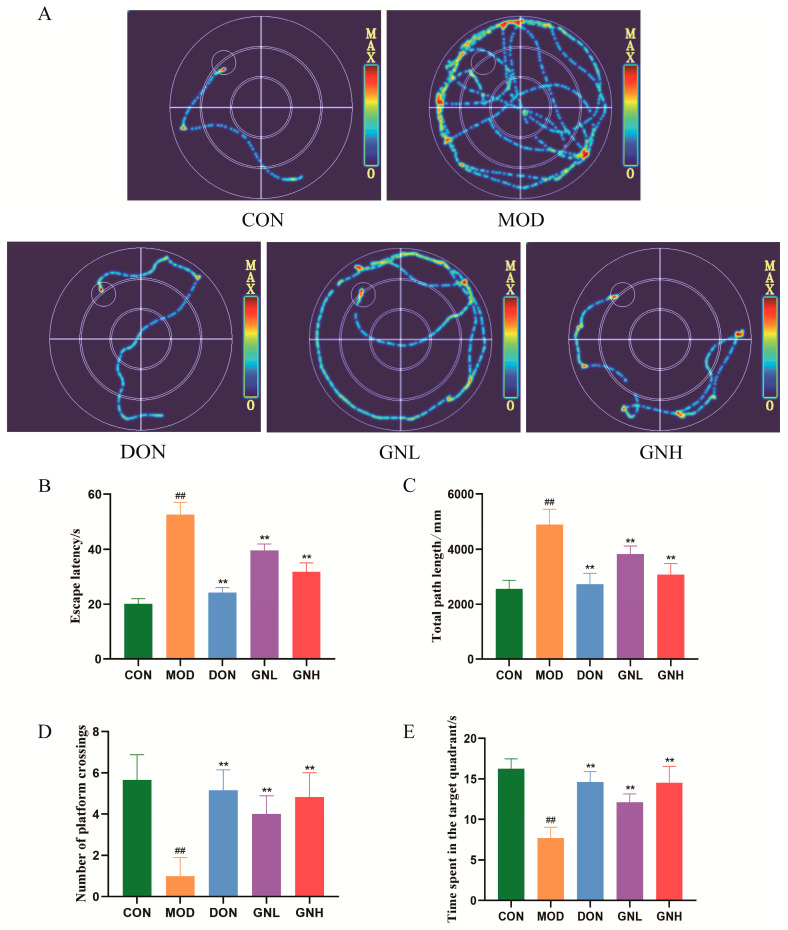
Effects of GN on learning and memory impairment of AD mice. (**A**) The swimming trajectories in mice. (**B**) Escape latency. (**C**) Total path length. (**D**) Number of platform crossings. (**E**) Time spent in the target quadrant. Data are presented as mean ± SD (*n* = 6). Significant differences are indicated by ## *p* < 0.01 vs. the CON group; ** *p* < 0.01 vs. the MOD group. One-way ANOVA followed by Tukey’s post hoc test (for (**B**–**E**)) was conducted to analyze data.

To evaluate the effect of GN on spatial learning and memory in AD mice, we first verified the successful establishment of the model using the MWM test. The swimming trajectories (Figure 2A) showed that mice in the model (MOD) group exhibited irregular and non-goal-oriented paths, failing to actively search for the platform and instead displayed peripheral circling behavior. As shown in Figure 2B–E, compared with the control (CON) group, mice in the MOD group exhibited significantly prolonged escape latency and increased total swimming distance, while the number of platform crossings and the time spent in the target quadrant were significantly decreased (*p* < 0.01). These results indicate that scopolamine successfully induced learning and memory dysfunction in the mice. As a positive control, intervention with the donepezil positive control (DON) group significantly reversed the aforementioned cognitive deficits in the MOD group (*p* < 0.01), with swimming trajectories that were more direct and goal-oriented, confirming the validity of the experimental system. More importantly, the GN intervention also demonstrated significant improvements. Compared with the MOD group, all measured parameters in both the low-dose GN (GNL) group and the high-dose GN (GNH) group were significantly improved (*p* < 0.01), with the GNH group exhibiting a more pronounced effect than the GNL group. In conclusion, GN effectively ameliorated the scopolamine-induced learning and memory impairment in AD mice.

### 2.3. Effects of GN on Cholinergic Function, Oxidative Stress, and Inflammation in AD Mice

**Figure 3:**
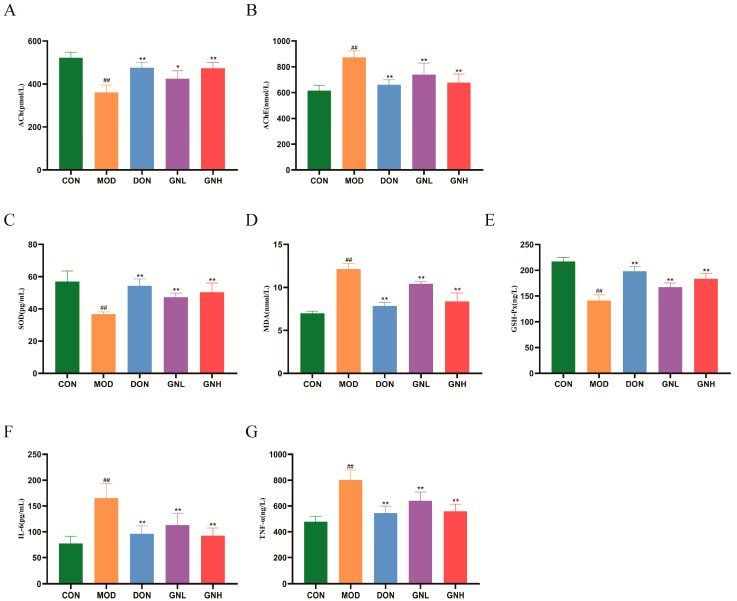
Effects of GN on Cholinergic Function, Oxidative Stress, and Inflammation in AD Mice. (**A**) ACh, (**B**) AChE, (**C**) SOD, (**D**) MDA, (**E**) GSH-PX, (**F**) IL-6, (**G**)TNF-α. Data are presented as mean ± SD (*n* = 6). Significant differences are indicated by ## *p* < 0.01 vs. the CON group; * *p* < 0.05, ** *p* < 0.01 vs. the MOD group. One-way ANOVA followed by Tukey’s post hoc test was conducted to analyze data.

The ELISA test results (Figure 3) indicate that compared with the CON group, the MOD group exhibited significantly decreased levels of acetylcholine (ACh), superoxide dismutase (SOD), and glutathione peroxidase (GSH-Px) (*p* < 0.01), while the levels of acetylcholinesterase (AChE), malondialdehyde (MDA), interleukin-6 (IL-6), and tumor necrosis factor-α (TNF-α) were significantly increased (*p* < 0.01). In contrast, intervention with both GNL and GNH significantly elevated the levels of ACh, SOD, and GSH-Px (*p* < 0.01 or *p* < 0.05) and significantly reduced the levels of AChE, MDA, IL-6, and TNF-α (*p* < 0.01) when compared to the MOD group. Notably, the GNH group demonstrated a superior effect to the GNL group in ameliorating these biochemical indicators. These findings suggest that GN exerts a significant ameliorative effect on scopolamine-induced AD mice through the multi-target regulation of the cholinergic, antioxidant, and inflammatory systems.

### 2.4. Effects of GN on the Brain Tissues Morphology in AD Mice

**Figure 4:**
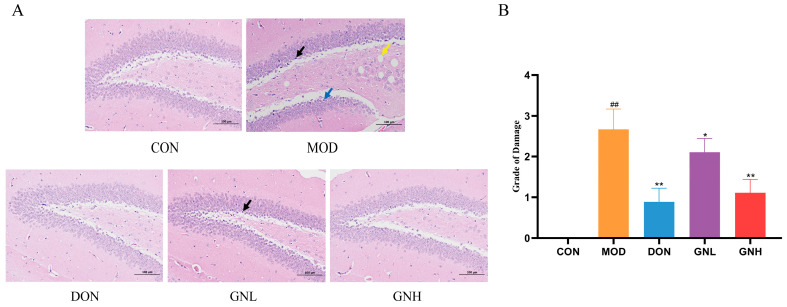
Effects of GN on the brain tissues morphology in AD mice. (**A**) Images of H&E staining. Black arrows indicate neuronal shrinkage; blue arrows indicate neuronal edema; yellow arrows indicate round vacuoles (Scale bar: 100 µm.) (**B**) Statistical analysis of the grade of neuronal damage of H&E staining. Data are presented as mean ± SD (*n* = 3). Significant differences are indicated by ## *p* < 0.01 vs. the CON group; * *p* < 0.05, ** *p* < 0.01 vs. the MOD group. One-way ANOVA followed by Tukey’s post hoc test was conducted to analyze data.

As shown in Figure 4A, the hippocampal region of mice in the CON group exhibited a rich population of neurons with intact morphological structures, arranged in a dense and orderly manner. The nuclei were large and round with clear nucleoli. In stark contrast, the hippocampal region of the MOD group presented significant neuronal damage, characterized by neuronal shrinkage, cell body pyknosis, blurred nucleo-cytoplasmic boundaries, cellular edema, and vacuolization. Compared with the MOD group, the treatment groups exhibited varying degrees of alleviation in neuronal damage. In the DON group, the neuronal damage was significantly alleviated, showing a morphological appearance that was close to that of the CON group, with a significant reduction in neuronal shrinkage. In the GNL group, neurons were arranged in a relatively dense manner with mostly intact morphology, though a few shrunken cells were still observed. The GNH group demonstrated a superior protective effect, exhibiting neurons with regular morphology and clear structures. As shown in Figure 4B, neuronal injury was significantly aggravated in the MOD group compared to the CON group (*p* < 0.01). However, neuronal injury in the DON, GNL, and GNH groups was significantly alleviated compared to the MOD group (*p* < 0.01, *p* < 0.05, and *p* < 0.01, respectively). These results confirm that GN effectively reversed the scopolamine-induced brain injury, with the GNH group exhibiting a superior intervention efficacy compared to the GNL group.

Although the initial pharmacological and histological evaluations included five groups to validate the model and assess dose-dependency, the results demonstrated that the intervention efficacy of GNH was significantly superior to that of the GNL. Therefore, to streamline the subsequent mechanistic investigation and focus on the most effective intervention strategy, we selected the CON, MOD, and GNH groups for follow-up experiments to further elucidate the underlying mechanism of action.

### 2.5. Effects of GN on the Gut Microbiota in AD Mice

#### 2.5.1. Effects of GN on Species Diversity

**Figure 5:**
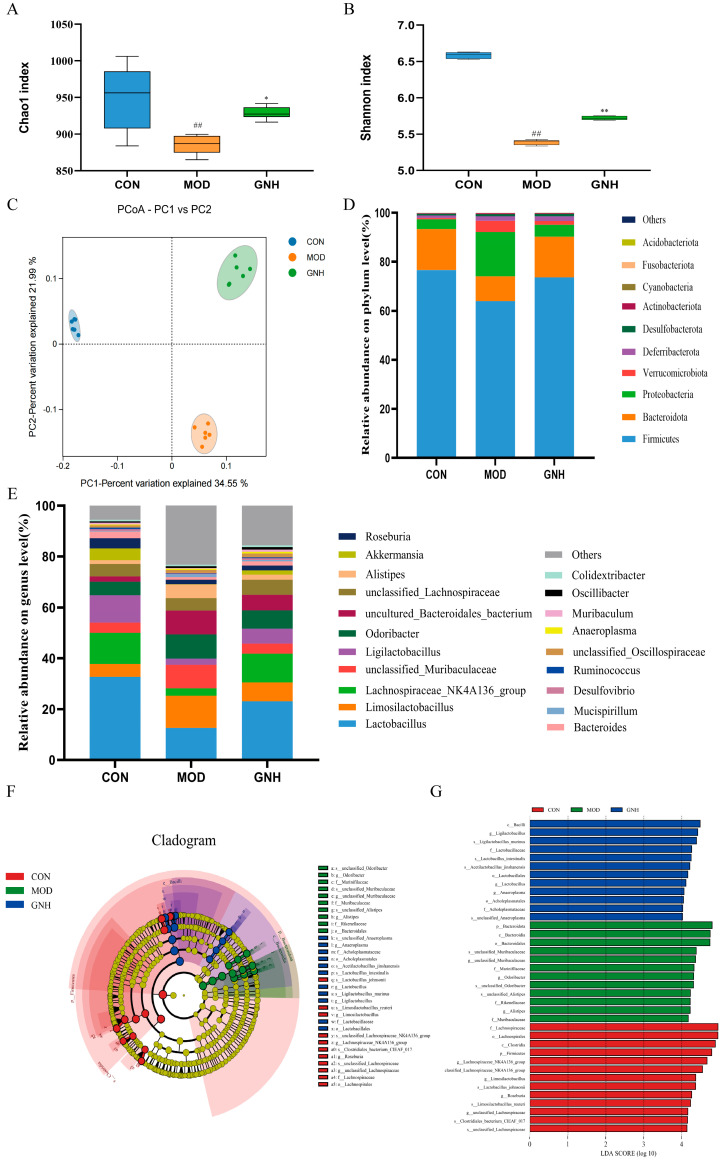
Effect of GN on the Gut Microbiota in AD Mice. (**A**) Chao1 index. (**B**) Shannon index. (**C**) PCoA. (**D**) Abundances of gut microbiota at the phylum level. (**E**) Abundances of gut microbiota at the genus level. (**F**) Cladogram from LEfSe analysis. (**G**) Bar plot of LDA scores (LDA > 4). Data are presented as mean ± SD (*n* = 6). Significant differences are indicated by ## *p* < 0.01 vs. the CON group; * *p* < 0.05, ** *p* < 0.01 vs. the MOD group. One-way ANOVA followed by Tukey’s post hoc test (for (**A**,**B**)) was conducted to analyze data.

Alpha diversity analysis was employed to assess the diversity within the microbial communities. The Chao1 index, which measures species richness (i.e., the number of species), and the Shannon index, which reflects species diversity influenced by both richness and evenness, were calculated. As shown in Figure 5A,B, the Chao1 and Shannon indices in the MOD group were significantly lower than those in the CON group (*p* < 0.01). In contrast, both indices in the GNH group were significantly higher than in the MOD group (*p* < 0.05 and *p* < 0.01, respectively). These results indicate that the gut microbiota in AD mice exhibited reduced richness and diversity, suggesting a state of dysbiosis. GN intervention effectively increased the microbial richness and diversity, potentially contributing to the restoration of a healthier gut ecosystem.

Beta diversity analysis was performed to evaluate the differences in microbial community structure and composition among the groups. The principal coordinates analysis (PCoA) results (Figure 5C) revealed that the microbial community of the MOD group formed a distinct cluster, clearly separated from those of the CON and GNH groups, indicating a significant shift in species composition. This finding confirmed that scopolamine-induced AD markedly altered the fecal microbiota of mice. GN intervention partially restored the diversity of the gut microbiota in AD mice, suggesting that it modulated the microbial community structure and counteracted the disruptive effects of the disease.

#### 2.5.2. Effects of GN on Species Composition

To visualize the taxonomic composition, stacked bar plots of the relative abundances were generated, displaying the top 10 phyla and top 20 genera in each sample.

At the phylum level (Figure 5D), the dominant bacterial phyla across the three groups were *Firmicutes*, *Bacteroidota*, and *Proteobacteria*. In the CON group, their relative abundances were 76.59%, 16.77%, and 3.90%, respectively. In contrast, the MOD group exhibited abundances of 63.98%, 10.11%, and 18.02% for these same phyla. Following GNH intervention, the composition shifted to 73.67%, 16.53%, and 4.85%, respectively. These results indicate that compared to the CON group, the MOD group displayed a significant decrease in the relative abundances of *Firmicutes* and *Bacteroidota*, accompanied by an increase in *Proteobacteria*. Notably, GNH intervention partially reversed these microbial dysbiosis at the phylum level.

Further analysis at the genus level revealed significant heterogeneity in the composition of dominant genera among the three groups, with varying relative abundances of shared taxa. Inter-group comparisons further elucidated these differences in species composition. As shown in Figure 5E, compared to the CON group, the MOD group exhibited a significant decrease in the relative abundance of *Lactobacillus* (by 61.54%), *Ligilactobacillus* (by 76.48%), *Akkermansia* (by 96.52%), and *Bacteroides* (by 61.95%). Conversely, the abundances of *Alistipes* and *Mucispirillum* increased by 71.58% and 83.16%, respectively. Following GNH administration, these trends were modulated. Compared to the MOD group, the GNH group showed an increased relative abundance of *Lactobacillus* (by 45.41%), *Ligilactobacillus* (by 57.56%), *Akkermansia* (by 89.99%), and *Bacteroides* (by 41.18%), while the abundances of *Alistipes* and *Mucispirillum* decreased by 61.47% and 54.62%, respectively. These results indicate that GNH increased the abundance of beneficial bacteria while reducing that of potentially harmful bacteria, demonstrating its potential beneficial properties. In summary, significant differences existed among the three groups in gut microbiota composition, dominant species, and their relative abundances, which may play a crucial role in disease progression and the underlying mechanisms of action.

#### 2.5.3. Linear Discriminant Analysis Effect Size (LEfSe) Analysis Results

To further identify differentially abundant taxa among the CON, MOD, and GNH groups, LEfSe analysis was performed (Figure 5F,G). A total of 37 taxa with an LDA score greater than 4 were identified. Among these, 13 taxa were enriched in the CON group, 12 in the MOD group, and 12 in the GNH group. At the phylum and genus levels, the CON group was enriched with *Firmicutes*, *Lachnospiraceae*_*NK4A136*_*group*, *Limosilactobacillus*, *Roseburia*, and *unclassified*_*Lachnospiraceae*. The MOD group showed enrichment of *Bacteroidota*, *unclassified*_*Muribaculaceae*, *Odoribacter*, and *Alistipes*. In contrast, the GNH group was enriched with *Ligilactobacillus*, *Lactobacillus*, and *Anaeroplasma*. These results indicate that GNH possesses the capacity to modulate the structure of the gut microbiota, thereby conferring potential benefits to the host.

### 2.6. Effects of GN on Serum Metabolomics in AD Mice

#### 2.6.1. Multivariate Data Analysis

**Figure 6:**
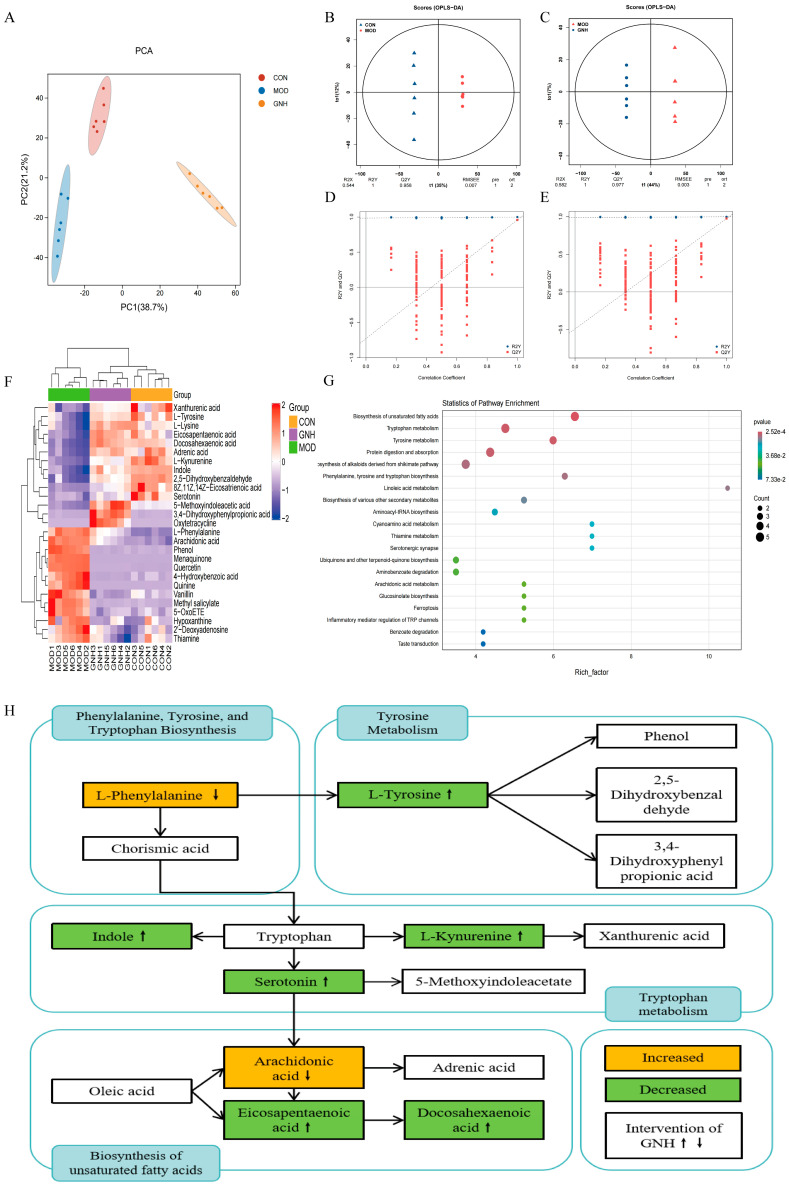
Effect of GN on Serum Metabolomics in AD Mice. (**A**) PCA. (**B**) OPLS-DA score plot of the CON and MOD groups. (**C**) OPLS-DA score plot of the MOD and GNH groups. (**D**) Permutation test of the OPLS-DA model for the CON and MOD groups. (**E**) Permutation test of the OPLS-DA model for the MOD and GNH groups. (**F**) Clustered heatmap of significantly different metabolites among the CON, MOD, and GNH groups. (**G**) KEGG pathway enrichment plot of differential metabolites. The color of the dots represents the *p*-value, with darker red indicating more significant enrichment. The size of the dots is proportional to the number of enriched differential metabolites. (**H**) Schematic diagram of the effect of GN on metabolic pathway dysregulation in AD mice. The color of the boxes represents the trend of change when comparing the MOD group to the CON group, where yellow indicates promotion and green indicates inhibition. The arrows on the right side of the metabolites within the boxes show the trend for the GNH group compared to the MOD group, with an upward arrow (↑) indicating promotion and a downward arrow (↓) indicating inhibition.

To initially assess the impact of GN on the overall serum metabolic profile of AD mice, principal component analysis (PCA) was first performed. The PCA score plot (Figure 6A) showed that samples within the CON, MOD, and GNH groups were closely clustered, indicating good intra-group reproducibility. In contrast, the three groups were distinctly separated on the plot, suggesting significant inter-group metabolic differences. To further identify the specific metabolites responsible for these differences, the orthogonal partial least squares discriminant analysis (OPLS-DA), a supervised pattern recognition method, was employed. The OPLS-DA model for the CON and MOD groups (Figure 6B) exhibited a clear separation, with model parameters R2Y and Q2Y of 1 and 0.958, respectively, demonstrating the model’s excellent explanatory and predictive power. Similarly, the MOD and GNH groups were also effectively discriminated (Figure 6C), with R2Y and Q2Y values of 1 and 0.977, respectively, indicating that GNH administration significantly altered the metabolic status of the AD mice. Furthermore, the results of the permutation test (Figure 6D,E) confirmed the robustness and reliability of the aforementioned OPLS-DA models, excluding the risk of overfitting. These validated models provided a solid foundation for the subsequent screening of potential differential metabolites.

#### 2.6.2. Differential Metabolite Screening and Metabolic Pathway Analysis

Differential metabolites were screened based on thresholds of fold change (FC) > 1, *p* < 0.05, and variable importance in projection (VIP) > 1. Through a comparative analysis of serum samples from the CON, MOD, and GNH groups, a total of 27 significantly differential metabolites were identified as potential biomarkers, primarily belonging to key metabolic categories such as amino acids, lipids, and vitamins. Compared to the CON group, the MOD group exhibited significant metabolic disturbances, with 13 metabolites upregulated and 14 downregulated. Notably, following GNH administration, the expression levels of these differential metabolites showed a trend of reverting towards the normal state of the CON group. The overall pattern of metabolic changes among the groups was intuitively visualized by a hierarchical clustering analysis heatmap (Figure 6F).

To further elucidate the biological functions regulated by GNH, Kyoto Encyclopedia of Genes and Genomes (KEGG) enrichment analysis was performed to map these differential metabolites to relevant metabolic pathways. The results (Figure 6G) indicated that the effects of GNH significantly impacted several key metabolic pathways, including the biosynthesis of unsaturated fatty acids, tryptophan metabolism, tyrosine metabolism, and phenylalanine, tyrosine and tryptophan biosynthesis. To gain deeper insights into the regulatory network of GNH, we constructed a metabolic pathway interaction network (Figure 6H) by integrating the aforementioned key pathways. This network clearly labeled the core metabolite nodes in each pathway, visually demonstrating the potential regulatory mechanisms of GNH intervention on these core metabolic pathways. This systematic visualization not only elucidated the multi-target effects of GN in modulating AD-associated metabolic disturbances but also provided a crucial theoretical basis and direction for subsequent investigations into the specific molecular mechanisms of GN in the context of AD.

### 2.7. Gut Microbiota–Metabolite Correlation Analysis

**Figure 7:**
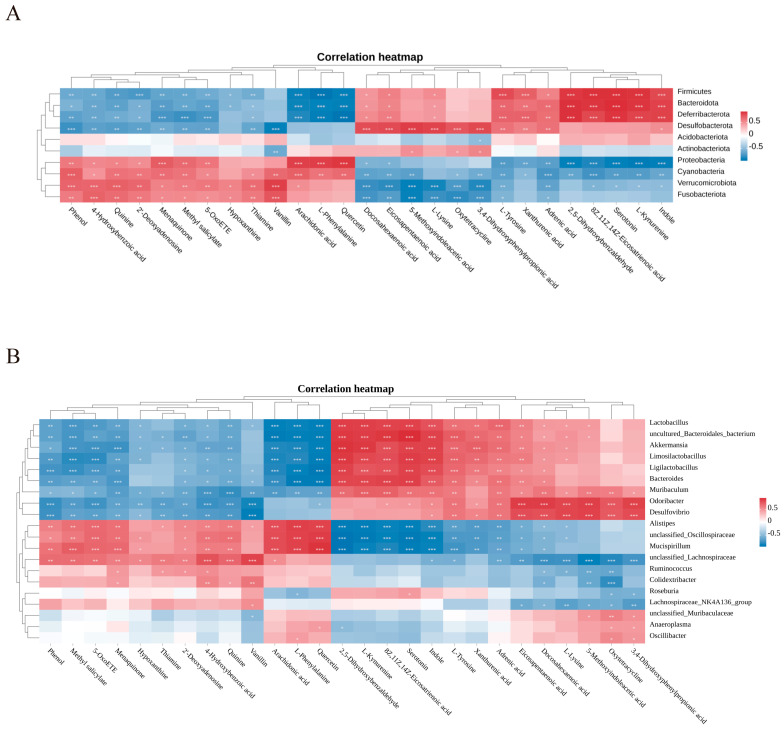
Gut Microbiota–Metabolite Correlation Analysis. (**A**) Correlation heatmap between phylum-level gut microbiota and differential metabolites. (**B**) Correlation heatmap between genus-level gut microbiota and differential metabolites. The colors represent the correlation coefficient. Red indicates a positive correlation, and blue indicates a negative correlation. * *p* < 0.05, ** *p*< 0.01, *** *p* < 0.001.

To further investigate the mechanism by which GN ameliorates cognitive function, we employed Spearman’s correlation coefficient to analyze the relationships between gut microbiota and serum differential metabolites at both the phylum and genus levels. A heatmap was used to visualize the correlations between OTU abundance of gut microbiota and the compositional changes of differential metabolites. As shown in Figure 7, significant correlations were observed between most gut microbiota and the differential metabolites.

At the phylum level (Figure 7A), indole, L-kynurenine, serotonin, L-tyrosine, eicosapentaenoic acid (EPA), and docosahexaenoic acid (DHA) showed significant positive correlations with *Firmicutes* and *Bacteroidota*, but significant negative correlations with *Proteobacteria*. In contrast, L-phenylalanine and arachidonic acid exhibited significant negative correlations with *Firmicutes* and *Bacteroidota*, and significant positive correlations with *Proteobacteria*. Furthermore, other phyla also demonstrated significant correlations with various metabolites. At the genus level (Figure 7B), DHA, EPA, L-tyrosine, indole, serotonin, and L-kynurenine were significantly positively correlated with *Lactobacillus*, *Akkermansia*, *Ligilactobacillus*, and *Bacteroides*, but significantly negatively correlated with *Alistipes* and *Mucispirillum*. Conversely, L-phenylalanine and arachidonic acid were significantly negatively correlated with *Lactobacillus*, *Akkermansia*, *Ligilactobacillus*, and *Bacteroides*, and significantly positively correlated with *Alistipes* and *Mucispirillum*. Other genera also showed substantial correlations with multiple metabolites.

These results suggest that GN may exert its neuroprotective effects by modulating the interactions between gut microbiota and serum metabolites, thereby ameliorating cognitive function in AD mice.

### 2.8. Effects of GN on Hippocampal ZO-1, Occludin, and Claudin-1 Protein Expression

**Figure 8:**
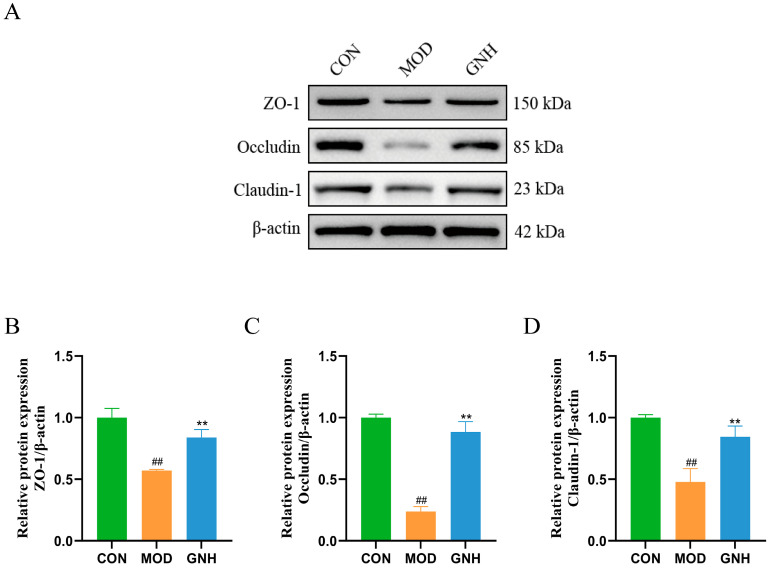
Effects of GN on Hippocampal ZO-1, Occludin, and Claudin-1 Protein Expression. (**A**) Individual protein bands and groupings. Relative protein expression levels of ZO-1 (**B**), Occludin (**C**), and Claudin-1 (**D**). Data are presented as mean ± SD (*n* = 3). Significant differences are indicated by ## *p* < 0.01 vs. the CON group; ** *p* < 0.01 vs. the MOD group. One-way ANOVA followed by Tukey’s post hoc test (for (**B**–**D**)) was conducted to analyze data.

This study evaluated the effect of GN on BBB integrity by detecting the expression of key tight junction proteins in mouse hippocampal tissue via Western blot. As shown in Figure 8, compared to the CON group, the protein expression levels of ZO-1, Occludin, and Claudin-1—key markers of the BBB—were significantly downregulated in the MOD group (*p* < 0.01), indicating that the AD model successfully induced BBB impairment. Conversely, GNH intervention effectively reversed this trend, significantly upregulating the expression of ZO-1, Occludin, and Claudin-1 (*p* < 0.01). These findings suggest that GNH may exert its neuroprotective effects by restoring the integrity of the compromised BBB.

## 3. Discussion

AD remains a formidable challenge, characterized by a complex interplay of pathological features including Aβ deposition, tau hyperphosphorylation, neuroinflammation, oxidative stress, and BBB damage [19,20]. Given the limitations of current clinical treatments, drug development is urgently needed. In this context, GN, with its neuroprotective properties, offers a novel and promising approach.

The anti-AD potential of GN stems from the synergistic effect of its various active components. Among these, ginsenosides exert beneficial effects by modulating multiple core pathological aspects of AD. For instance, ginsenosides Rg1 and Rb1 mediate neuroprotection primarily through anti-inflammatory, anti-apoptotic, and anti-oxidative stress pathways [21,22]. Furthermore, ginsenoside Rc contributes to the amelioration of the AD pathophysiological state by inhibiting the activities of AChE and butyrylcholinesterase (BChE) [23]. Another signature component, nattokinase, exhibits significant neuroprotective functions by effectively alleviating BBB dysfunction and mitigating neuroinflammatory responses, which contributes to the enhancement of cognitive abilities [24]. Concurrently, the soy isoflavones present exert potent antioxidant, anti-apoptotic, and anti-inflammatory effect [25]. These actions collectively protect the structural and functional integrity of the cerebrovascular system, thereby improving cognitive performance in AD. The core innovation of GN resides in the synergistic integration of its primary constituents—ginsenosides, nattokinase, and soy isoflavones—achieved through a sophisticated bio-fermentation methodology. This methodology yields a formulation whose efficacy is greater than the sum of its parts, moving beyond the limitations of a simple additive mixture. Consequently, it provides a more rigorous scientific foundation for developing preventive and disease-modifying strategies against AD. This study elucidated GN’s neuroprotective mechanism in AD mice using a multi-level approach. We confirmed cognitive benefits via the MWM test, ELISA, and H&E staining and explored the underlying mechanism by integrating 16S rRNA sequencing for gut microbiota, metabolomics for serum metabolites, and protein analysis to assess BBB integrity, revealing a comprehensive gut–brain axis regulatory network.

This study demonstrates that GN exerts a significant neuroprotective effect in AD mice, primarily characterized by the amelioration of spatial learning and memory deficits. At the histopathological level, GN intervention effectively mitigated damage in the hippocampus, evidenced by an increased neuronal count and the restoration of normal morphology. On a biochemical level, GN exhibited multi-targeted synergistic regulatory effects. Specifically, GN modulated the cholinergic system by upregulating ACh levels and inhibiting AChE activity; enhanced antioxidant capacity by increasing SOD and GSH-Px activities while reducing MDA accumulation; and exerted anti-inflammatory effects by suppressing the expression of IL-6 and TNF-α. Therefore, the neuroprotection conferred by GN is not attributable to a single pathway, but rather arises from the synergistic integration of cholinergic regulation, enhanced antioxidant defense, and anti-inflammatory actions. Notably, these beneficial effects exhibited a clear dose-dependent relationship, with the GNH group showing the most pronounced regulatory outcomes.

Our findings indicate that oxidative stress and neuroinflammation interact closely, mutually exacerbating AD progression. GN intervention significantly enhanced the activities of antioxidant enzymes SOD and GSH-Px while reducing the MDA levels, indicating the effective mitigation of oxidative stress. Concurrently, GN suppressed pro-inflammatory cytokines IL-6 and TNF-α. Existing evidence suggests that oxidative stress can trigger neuroinflammation through pathways such as nuclear factor-κB (NF-κB) activation, which in turn promotes further reactive oxygen species (ROS) generation—creating a vicious cycle that accelerates Aβ deposition, tau hyperphosphorylation, and neuronal apoptosis, ultimately driving cognitive decline [26,27]. In this study, the simultaneous modulation of both oxidative and inflammatory markers by GN suggests a disruption of this detrimental feedback loop, potentially slowing neurodegenerative progression. This synergistic action supports GN as a multi-target strategy capable of improving not only biochemical profiles but also influencing key pathological pathways involved in the long-term course of AD.

Gut dysbiosis is a key driver of neuroinflammation in AD [28]. Alterations in gut microbiota abundance disrupt normal gut–brain communication by affecting enteric nervous signaling and inducing systemic inflammation, thereby promoting AD progression [29]. In the present study, at the phylum level, mice in the MOD group exhibited a significant decrease in the relative abundance of *Firmicutes* and *Bacteroidota*, accompanied by a marked increase in *Proteobacteria*, a finding consistent with previous reports [30,31]. Such an imbalance reduces the generation of beneficial metabolites with neuroprotective properties, such as short-chain fatty acids (SCFAs), while simultaneously exacerbating gut barrier dysfunction and systemic inflammation, thereby promoting AD pathological processes via the microbiota–gut–brain axis [32,33]. Notably, GN intervention effectively modulated and reversed this dysbiosis. This suggests that reshaping gut microbial homeostasis to alleviate neuroinflammation is a key mechanism through which GN exerts its beneficial effects. At the genus level, mice in the MOD group exhibited a significant reduction in the relative abundance of several genera with potential neuroprotective properties, including *Lactobacillus*, *Ligilactobacillus*, *Akkermansia*, and *Bacteroides*. In contrast, inflammation-associated genera, such as *Alistipes* and *Mucispirillum*, were significantly enriched. The beneficial genera contribute to neuroprotection through various mechanisms: *Lactobacillus* mitigates AD neurodegeneration by modulating γ-aminobutyric acid (GABA) and promoting the production of SCFAs [34,35]; *Ligilactobacillus* positively influences brain function by enhancing gut barrier integrity, modulating immune responses, and alleviating stress [36,37]; *Akkermansia* alleviates peripheral and central inflammation by strengthening the gut barrier and promoting butyrate producers, linking its depletion to key AD pathologies [38]. *Bacteroides* is involved in key metabolic activities and is closely associated with neurodevelopment and depression. Conversely, the pro-inflammatory genera exacerbate AD pathology [39,40]. *Alistipes* and *Mucispirillum* promote chronic inflammation via the gut–brain axis [41,42]. Our study demonstrates that GN intervention reverses this dysbiosis, restoring beneficial bacteria and suppressing pro-inflammatory ones. This microbiota remodeling is a central mechanism for GN’s cognitive benefits, as it disrupts gut–brain axis inflammatory signaling and reduces systemic and neuroinflammation.

Our metabolomics analysis revealed that GN intervention significantly modulated four key metabolic pathways in AD mice: the biosynthesis of unsaturated fatty acids, tryptophan metabolism, tyrosine metabolism, and the biosynthesis of phenylalanine, tyrosine, and tryptophan. Amino acid dysregulation is a core pathological feature of AD [43]. Tryptophan metabolites, generated via the kynurenine, serotonin, and indole derivative pathways, collectively regulate neurological function, immune homeostasis, and gut–brain axis communication [44]. Concurrently, the phenylalanine-tyrosine pathway, a source of precursors for catecholamine neurotransmitters, is implicated in neurotoxicity when dysregulated [45]. We found that GN intervention effectively ameliorated this AD-associated amino acid imbalance by significantly decreasing the serum levels of abnormally elevated L-phenylalanine while increasing the levels of L-tyrosine, L-kynurenine, serotonin, and indoles. Unsaturated fatty acids are vital for brain function. ω-3 polyunsaturated fatty acids (PUFAs), such as DHA and EPA, possess well-established neuroprotective properties, including maintaining BBB integrity [46]. In contrast, ω-6 PUFAs, like arachidonic acid (AA), primarily mediate pro-inflammatory and neurotoxic processes [47,48]. Our study demonstrated that GN intervention elevated DHA and EPA levels while reducing AA levels, thereby shifting the lipid metabolic profile from a pro-inflammatory to an anti-inflammatory and neuroprotective state. Collectively, these four interconnected pathways form a core metabolic network through which GN exerts its multi-targeted neuroprotective effects. These findings provide robust metabolomic evidence for the efficacy of GN in ameliorating AD and offer clear directions for future research into its molecular targets and treatment strategies.

To elucidate the molecular mechanisms, this study integrated 16S rRNA sequencing with metabolomics, revealing that GN ameliorates AD by modulating the microbiota–metabolite axis. We found that the alterations in the microbial structure of *Firmicutes*, *Bacteroidota*, and *Proteobacteria* were closely associated with the levels of differential serum metabolites. Meanwhile, our analysis indicated that GN effectively reversed gut dysbiosis. Specifically, *Lactobacillus* promoted the conversion of tryptophan to serotonin to enhance cognition [49]; *Akkermansia* protected neurons by regulating ω-3 PUFAs and maintaining BBB integrity [50]; *Ligilactobacillus* produced indole-3-lactic acid to inhibit neuroinflammation [51,52]; and *Bacteroides* influenced tyrosine metabolism, thereby modulating dopamine synthesis and cognitive function [53,54]. Concurrently, suppressing pro-inflammatory genera like *Alistipes* and *Mucispirillum* prevented their detrimental effects on serotonin synthesis and gut barrier function [55]. These microbial changes subsequently influenced four key metabolic pathways. In summary, the neuroprotective effects of GN arise from a systemic remodeling of the microbiota–metabolite axis, which optimizes the host’s metabolic milieu by elevating neuroprotective metabolites and reducing pro-inflammatory factors, ultimately ameliorating cognitive impairment in AD mice.

BBB is a critical structure for maintaining homeostasis within the central nervous system, primarily constituted by brain microvascular endothelial cells and their tight junctions. Among these, ZO-1, Occludin, and Claudin-1 are core proteins of the tight junction complex, collectively determining the integrity and selective permeability of the BBB [56]. In the pathological progression of AD, factors such as Aβ deposition, Tau protein abnormalities, and neuroinflammation can lead to BBB dysfunction. This manifests as a significant downregulation of ZO-1, Occludin, and Claudin-1 expression, resulting in increased barrier permeability, the influx of peripheral harmful substances into the brain, and the exacerbation of neurodegeneration [57]. In the present study, we observed that the protein expression levels of ZO-1, Occludin, and Claudin-1 were markedly reduced in the hippocampal tissue of MOD mice, which is consistent with the pathological features of BBB dysfunction. Conversely, following GN intervention, the expression of these proteins was significantly upregulated. This suggests that GN may exert its neuroprotective effects by restoring BBB integrity. These findings not only provide a crucial molecular mechanism for the amelioration of AD pathology by GN but also further corroborate that targeting BBB tight junction proteins represents a vital strategy for AD treatment.

In summary, our study demonstrates that GN exerts potent anti-AD effects through a synergistic, multi-target mechanism. By reshaping the gut microbiota and modulating associated metabolites, GN restores metabolic homeostasis, reinforces the BBB, and suppresses neuroinflammation, offering a novel and comprehensive intervention approach for AD.

## 4. Materials and Methods

### 4.1. Chemicals and Reagents

*Panax ginseng* (batch no. 230302) was obtained from Antu City Anxing Decoction Pieces Co., Ltd. (Antu, China). The bacterial strain *Bacillus subtilis* (CICC 10023) was procured from the China Center of Industrial Culture Collection (CICC; Beijing, China). Scopolamine hydrobromide (DSTDQ002703) was purchased from Chengdu Dest Biotechnology Co., Ltd. (Chengdu, China), and donepezil hydrochloride tablets (batch no. 2303148) were supplied by Eisai China Inc. (Shanghai, China).

### 4.2. Preparation of GN and Determination of Its Main Component Content

#### 4.2.1. Preparation of GN

Soybeans and ginseng were initially weighed at a 6:1 mass ratio. A ginseng extract solution was prepared by decocting the ginseng with distilled water twice for 1 h per session and combining the filtrates. The soybeans were then soaked in this extract solution at a 1:1.2 (g:g) ratio until fully absorbed. The resulting mixture was sterilized by autoclaving at 121 °C for 30 min to create the fermentation substrate. After cooling, the substrate was inoculated with *Bacillus subtilis* at an inoculum size of 6.2% (*w*/*w*), mixed thoroughly, and subsequently incubated at 34 °C for 37.2 h. The crude product obtained was then dried, pulverized, and sieved through a No. 3 mesh to yield the final GN powder.

#### 4.2.2. Quantification of Major Bioactive Constituents in GN

##### Nattokinase Activity Assay

The crude nattokinase extract was prepared by adding 1 g of GN powder to 5 mL of sterile saline. The mixture was thoroughly extracted for 2 h and then centrifuged at 8000 r/min for 15 min at 4 °C. The resulting supernatant was collected as the crude extract. The nattokinase activity was determined using the agarose fibrin plate method [58], with urokinase serving as the standard. A series of urokinase standard solutions were prepared with activities of 100, 200, 400, 800, and 1600 IU/mL for the calibration curve.

##### HPLC Analysis of Soy Isoflavones and Ginsenosides

To prepare the sample for chemical analysis, 4.0 g of GN powder was mixed with 20 mL of methanol (5 volumes) and subjected to ultrasonic extraction for 1 h. The mixture was subsequently filtered, and the filtrate was concentrated to obtain the GN extract. The contents of soy isoflavones and ginsenosides were quantified by HPLC. Standard solutions of daidzin, glycitin, genistin, daidzein, genistein, ginsenoside Rg1, ginsenoside Rb1, and ginsenoside Rc were prepared in methanol at concentrations of 4.0320, 0.4008, 4.0120, 0.2040, 0.3000, 2.0200, 1.0160, and 0.5040 mg/mL, respectively.

Chromatographic analysis was performed on a Waters XBridge C18 column (250 mm × 4.6 mm, 5 μm) (Waters, Milford, CT, USA). The mobile phase consisted of acetonitrile (A) and a 0.1% aqueous solution of phosphoric acid (B), delivered at a flow rate of 1.0 mL/min with the following gradient program: 0.01–5 min, 90% B; 5–10 min, 90% B to 85% B; 10–30 min, 85% B to 82% B; 30–40 min, 82% B to 80% B; 40–70 min, 80% B to 74% B; 70–80 min, 74% B to 71% B; 80–95 min, 71% B; and 95–120 min, 71% B to 60% B. The analysis was conducted with the column temperature set to 30 °C, and the sample injection volume was 5 μL. Detection was performed at dual wavelengths: 254 nm for daidzin, glycitin, genistin, and daidzein (10–50 min) and genistein (65–75 min); and 203 nm for ginsenoside Rg1 (50–60 min), ginsenoside Rb1, and ginsenoside Rc (100–110 min).

### 4.3. Animals and Experimental Design

Specific pathogen-free (SPF) healthy male ICR mice (20 ± 2 g) were purchased from Liaoning Changsheng Biotechnology Co., Ltd (Shenyang, China). [license No. SCXK (Liao) 2020-0001]. All animal procedures were approved by the Animal Experimentation Ethics Committee of Changchun University of Chinese Medicine (approval No. 2024276).

**Figure 9:**
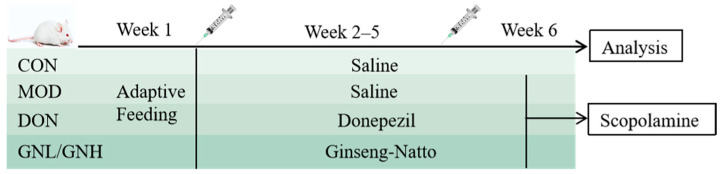
Animal experiment process.

All mice were acclimatized for one week prior to the experiment. They were housed in an animal room with an independent ventilation system, where the ambient temperature was maintained at (24 ± 2)°C and relative humidity at (55 ± 5)%. Throughout the study, all mice had ad libitum access to food and water. Subsequently, the mice were randomly divided into five groups (*n* = 6): the control group (CON), the model group (MOD), the donepezil positive control group (DON, 0.65 mg/kg), the low-dose GN group (GNL, 2.5 g/kg), and the high-dose GN group (GNH, 5.0 g/kg). Both the CON and MOD groups were administered the same volume of normal saline via gavage daily. From week 6, the AD model was induced in all groups except the CON group via intraperitoneal injection of scopolamine (2 mg/kg, prepared in normal saline). The CON group received an intraperitoneal injection of an equal volume of normal saline. Subsequently, behavioral experiments were conducted on the mice. The experimental procedure is illustrated in Figure 9.

After the completion of the behavioral experiments, all mice were euthanized. Blood samples and brain tissues were harvested. The brains were then divided along the midline, and the hippocampus was meticulously dissected from one part of the tissue. Serum was obtained by centrifuging the blood at 3000 r/min for 10 min at 4 °C. Using sterile instruments, the abdominal cavity was opened to expose the internal organs, the ileum was isolated, and fecal samples were collected. All collected samples were immediately stored at −80 °C for subsequent analysis. All procedures were performed under strict aseptic conditions.

### 4.4. MWM Test

The learning and memory abilities of the mice were assessed using the MWM test, which consisted of three phases: an acclimation trial, an acquisition trial, and a probe trial. The MWM apparatus was a circular pool divided into four equal quadrants, with a hidden platform submerged in the third quadrant. On day 1, the acclimation trial was conducted to allow the mice to adapt to the apparatus. From days 2 to 6, the acquisition trial was performed. In each trial, a mouse was given 60 s to find the hidden platform and was required to remain on it for 10 s. On the final day of this phase (day 6), the swimming trajectory, escape latency, and total path length were recorded as key indicators. On day 7, the probe trial was conducted to evaluate spatial memory. The hidden platform was removed, and each mouse was allowed to swim freely for 60 s. The number of platform crossings over the original platform location and the time spent in the target quadrant were recorded.

### 4.5. ELISA Analysis

The mouse brain tissues were thoroughly homogenized, and the homogenates were subjected to centrifugation at 4 °C and 3000 r/min for 20 min. The supernatant obtained after this centrifugation step was utilized for the quantification of various biomarkers with commercially available ELISA kits (Jiangsu Meimian Industrial Co., Ltd., Yancheng, China). These included markers of the cholinergic system: ACh and AChE; markers of the antioxidant system: SOD, MDA, and GSH-Px; and markers of the inflammatory system: IL-6 and TNF-α. All assays were performed strictly in accordance with the manufacturer’s instructions.

### 4.6. H&E Staining

Brain tissues were initially fixed in 4% paraformaldehyde, followed by dehydration using an ethanol gradient. The samples were then embedded in paraffin and cut into 4-μm-thick sections, which were subjected to H&E staining. Morphological alterations were examined via light microscopy. The severity of hippocampal neuronal injury in different groups of mice was quantified using semi-quantitative analysis. The scoring system is presented in Table 2 [59].

### 4.7. 16S rRNA Gene Sequencing Analysis

Total genomic DNA was extracted from the collected mouse fecal samples using the VAMNE Stool/Soil DNA Extraction Kit (Nanjing Vazyme Biotechnology Co., Ltd., Nanjing, China), following the manufacturer’s instructions. The integrity of the extracted DNA was examined using 1.8% agarose gel electrophoresis. Furthermore, the DNA concentration was quantified with a NanoDrop 2000 UV–Vis spectrophotometer (Thermo Scientific, Wilmington, NC, USA). Subsequently, the V1–V9 hypervariable regions of the 16S rRNA gene were amplified by PCR with specific primers. After quantification, the amplicons were pooled in equimolar amounts to construct the sequencing library. Finally, sequencing was performed on the PacBio Sequel II platform (Beijing Biomarker Technologies Co., Ltd., Beijing, China).

### 4.8. Serum Metabolomics Analysis

Metabolomic profiling was conducted via a liquid chromatography-tandem mass spectrometry (LC-MS/MS) platform. This setup included an Ultimate 3000 high-performance liquid chromatograph connected to an Orbitrap Exploris 480 high-resolution mass spectrometer. Chromatographic separation was achieved on a Waters Acquity UPLC HSS T3 column (1.8 μm, 2.1 mm × 100 mm). The mobile phase comprised 0.1% formic acid in water (Solvent A) and an equal concentration of formic acid in acetonitrile (Solvent B), with an injection volume of 1 μL. MS/MS data were collected using a data-dependent acquisition mode. We set the full scan mass range from *m*/*z* 67 to 1000. For the ESI source, the spray voltage was adjusted to 3500 V for positive ions and −2500 V for negative ions. The flow rates for sheath gas, auxiliary gas, and sweep gas were 50, 10, and 1 arbitrary units, respectively. The ion transfer tube was held at 325 °C, while the vaporizer was heated to 350 °C. Following acquisition with MassLynx V4.2, the raw data were analyzed using Progenesis QI V3.1 software to perform peak extraction and alignment. Metabolite identification was subsequently performed by searching against the mzCloud, mzVault, and ChemSpider databases. To assess experimental reproducibility and data quality, PCA was initially conducted on the normalized metabolomics data. Following this, compounds were annotated using databases such as KEGG and Human Metabolome Database (HMDB). The screening of differential metabolites integrated both univariate and multivariate statistical analyses, combining the FC, *p*-values from the *t*-test, and VIP scores from the OPLS-DA model. The reliability of the OPLS-DA model was confirmed through a permutation test. Ultimately, key differential metabolites were selected based on the thresholds of FC > 1, *p* < 0.05, and VIP > 1. These metabolites were then subjected to KEGG pathway enrichment analysis to elucidate their potential biological functions.

### 4.9. Gut Microbiota–Metabolite Correlation Analysis

To investigate the potential mechanism by which GN ameliorates metabolic disorders in AD mice through the regulation of the gut microbiota, a correlation analysis between the gut microbiota and differential metabolites was conducted. Based on 16S rRNA sequencing, the dominant bacterial taxa at the phylum level (top 10 in relative abundance) and the genus level (top 20 in relative abundance) were identified in the CON, MOD, and GNH groups. Concurrently, metabolomics was employed to identify key differential metabolites among the three groups, and KEGG pathway enrichment analysis was performed to determine their principal metabolic pathways. To establish the microbiota–metabolite association network, we analyzed the correlation between the previously identified dominant bacterial taxa and key differential metabolites using Spearman’s correlation coefficient. The results were visualized in a heatmap to reveal their potential interactions.

### 4.10. Western Blot Analysis

The expression levels of the BBB proteins, including ZO-1, Occludin, and Claudin-1, in mouse hippocampal tissues were assessed by Western blotting. The primary antibodies used were as follows: anti-ZO-1 (21773-1-AP, Proteintech, Wuhan, China), anti-β-Actin (66009-1-Ig, Proteintech, Wuhan, China), anti-Occludin (ab216327, Abcam, Cambridge, UK), and anti-Claudin-1 (bsm-60249R, Bioss, Beijing, China).

To isolate total protein, homogenized hippocampal tissues were lysed in RIPA buffer. After centrifugation at 12,000 rpm for 15 min at 4 °C, the supernatants were harvested. Protein levels were quantified using the BCA method. Subsequently, the samples were subjected to SDS-PAGE for separation and then transferred onto PVDF membranes, which were blocked with 5% non-fat dry milk. Subsequently, the membranes were incubated overnight at 4 °C with primary antibodies against ZO-1, Occludin, Claudin-1, and the internal control β-Actin, followed by incubation with a horseradish peroxidase (HRP)-conjugated secondary antibody for 1 h at room temperature. Following every incubation, the membranes underwent thorough washing with TBST. Ultimately, protein visualization was achieved via enhanced chemiluminescence (ECL) reagent, and the resulting signals were captured with a chemiluminescence imaging system (SH-523; Hangzhou Shenhua Technology Co., Ltd., Hangzhou, China). Quantitative analysis was performed using ImageJ software (version 1.46).

### 4.11. Statistical Analysis

All data are presented as the mean ± standard deviation (SD). Statistical analysis and graphical plotting were performed using GraphPad Prism 9.5 software (GraphPad Software, Inc., Boston, MA, USA). For comparisons among multiple groups, one-way analysis of variance (ANOVA) was applied, followed by Tukey’s post hoc test to identify specific differences between groups. The normality of the data distribution was verified using the Shapiro–Wilk test. Statistical significance was defined as *p*-values less than 0.05.

## 5. Conclusions

In conclusion, GN ameliorates cognitive impairment by targeting the microbiota–metabolite axis. GN reverses gut dysbiosis, modulates key metabolic pathways, and restores BBB integrity via upregulation of tight junction proteins. This gut–metabolite–brain regulatory network underpins the intervention potential of GN as a novel multi-target strategy for AD.

## Figures and Tables

**Table 1:** Chemical components of GN.

Index Component	Standard Curve	R^2^	Content
Nattokinase	Y = 0.1688 X + 225.38	0.9961	6645.3673 ± 109.6755 IU/g
Daidzin	Y = 15,120,363 X + 837109	0.9995	0.9571 ± 0.0070 mg/g
Glycitin	Y = 16,289,055 X − 53428	0.9996	0.1160 ± 0.0055 mg/g
Genistin	Y = 21,451,262 X − 548713	0.9996	0.9716 ± 0.0166 mg/g
Daidzein	Y = 30,171,512 X − 31744	0.9995	0.0332 ± 0.0015 mg/g
Genistein	Y = 31,608,972 X − 92168	0.9997	0.0433 ± 0.0019 mg/g
Ginsenoside Rg1	Y = 1,729,219 X − 38244	0.9996	0.3261 ± 0.0077 mg/g
Ginsenoside Rb1	Y = 1,080,774 X − 11421	0.9997	0.2729 ± 0.0044 mg/g
Ginsenoside Rc	Y = 1,318,088 X − 10598	0.9995	0.0942 ± 0.0022 mg/g

**Table 2:** Quantitative Scoring of Neuronal Damage.

Score	Degree of Injury	Morphological Characteristics
0	Normal	The neurons displayed intact morphology and orderly arrangement, with clearly visible nuclei and distinct nucleoli.
1	Mild	A small number of neurons were damaged (<25%), characterized by slight nuclear pyknosis, with the cells arranged in a basically orderly manner.
2	Moderate	A portion of neurons were damaged (25–50%), characterized by partial nuclear pyknosis and karyolysis, with disordered cell arrangement.
3	Severe	A large number of neurons were damaged (>50%), characterized by widespread nuclear pyknosis and nuclear fragmentation, indistinct cell outlines, and marked cellular edema and vacuolization.

## Data Availability

The original contributions presented in this study are included in the article. Further inquiries can be directed to the corresponding authors.

## Abbreviations

The following abbreviations are used in this manuscript:

ACh	Acetylcholine
AChE	Acetylcholinesterase
AD	Alzheimer’s disease
ANOVA	One-way analysis of variance
BBB	Blood–brain barrier
BChE	Butyrylcholinesterase
ELISA	Enzyme-linked immunosorbent assay
ESI	Electrospray ionization
FC	Fold change
GN	Ginseng-Natto composite fermentation products
GSH-Px	Glutathione peroxidase
H&E	Hematoxylin and Eosin
HMDB	Human Metabolome Database
HPLC	High-performance liquid chromatography
IL-6	Interleukin-6
KEGG	Kyoto Encyclopedia of Genes and Genomes
LC-MS/MS	Liquid chromatography-tandem mass spectrometry
LEfSe	Linear discriminant analysis Effect Size
MDA	Malondialdehyde
MWM	Morris Water Maze
NF-κB	Nuclear factor-κB
OPLS-DA	Orthogonal partial least squares discriminant analysis
PCA	Principal Component Analysis
PCoA	Principal Coordinates Analysis
ROS	Reactive oxygen species
SCFAs	Short-chain fatty acids
SOD	Superoxide dismutase
SPF	Specific pathogen-free
